# Protocol for the Implementation and Evaluation of a Mindfulness-Based Intervention for Caregivers of Children with Mental Disorders in a Clinical Setting

**DOI:** 10.3390/ijerph182010777

**Published:** 2021-10-14

**Authors:** Ana Pizarro-Carmona, Sofía Baena, Patricia Jiménez, Lucía Jiménez

**Affiliations:** 1 Faculty of Psychology, University of Seville, 41018 Seville, Spain; anapizcar@alum.us.es (A.P.-C.); luciajimenez@us.es (L.J.); 2 Andalusian Health Service, Jerez University Hospital, 11407 Jerez de la Frontera, Spain; mbihumanizacion.sc.sspa@juntadeandalucia.es

**Keywords:** mindfulness, evaluation protocol, positive parenting, stress, mental health disorders, evidence-based program

## Abstract

Being a parent is complicated in typical circumstances, with a great psychological impact as well as feelings and experiences of great intensity. This impact is greater in families in vulnerable situations, such as those with children with mental health problems, receiving treatment in a clinical setting. Due to these challenges, parenting in these circumstances is often accompanied by experiences of stress. An approach that has shown evidence of effectiveness in mitigating the negative impact of stress is mindfulness-based interventions, including the Mindfulness-Based Stress Reduction intervention program. The Mindfulness-Based Stress Reduction intervention program is designed as a psychoeducational, instructional, multimodal, and structured program whose main objective is to provide strategies for the management, coping, and awareness of stress in order to reduce it. In this paper, a protocol for the implementation and evaluation of the original Mindfulness-Based Stress Reduction intervention program with the added positive parenting component is presented, in order to systematize the incorporation of a parenting component in the Mindfulness-Based Stress Reduction intervention program, analyze its effectiveness for parents whose children have mental health problems (in terms of stress, mindfulness, emotional intelligence, general health, and parental role), explore the mechanisms of change operating in this intervention as perceived by the participants, and examine the application of acquired strategies to daily life.

## 1. Introduction

### 1.1. Parenting Children with Mental Health Disorders

Parenting is a task that requires time, effort, support, and learning. Everyday childcare involves challenges related to the provision of care to promote the optimal development of children, always taking into account the circumstances in which each family is immersed. Due to these challenges, parenting is often accompanied by experiences of stress which, in turn, has an effect on different facets of their daily life [1,2]. Although sometimes these stressful experiences can be balanced by the benefits and satisfactions of parenting, these negative emotions can persist and even intensify when there is an overflow of demands that cannot be met. Certain conditions or difficulties in the family, such as having a child with a disability or mental health disorder, increase this overflow of demands and, thus, challenges the existing resources that the families have to meet these demands adequately. Some of the additional demands that these parents face include the establishment of relationships and coordination with multiple professionals and services, lack of information about the resources available, the navigation of the resource and intervention system and the diagnosis, grief over the diagnosis, and feelings of inadequacy and incompetence in their parental role [3,4]. In particular, parents of adolescents with mental health disorders also face challenges related to the stigma associated with these disorders, internal and external guilt, lack of understanding of the symptoms and, in some cases, lack of involvement in the therapeutic process of their children, often feeling left out of the decision-making process [4]. These additional demands are often associated with an increase in the levels of perceived stress [5], along with other negative health-related symptoms, such as anxiety [6]. Moreover, studies indicate that parenting in this context could involve a deterioration of family relationships, including marital functioning, co-parenting, and family climate [6]. In this population, it is known that some of the most important problems that have been observed are difficulties in self-regulation, which limits the suitable management of conflicts and of the new stressful situations that they face every day [7].

There is evidence on how these negative symptoms related to the health of caregivers have a direct impact on childcare, the daily lives of families, and the wellbeing of children [8]. Fortunately, parents have access to resources and supports that minimize the effects of negative symptoms through different mechanisms. One of the support modalities that have proven to be helpful in alleviating these effects, and in acquiring tools that can help in the exercise of parenting, is the practice of meditation through awareness [9,10,11,12].

### 1.2. Mindfulness as a Parenting Resource

*Mindfulness* is a discipline that has its origin in Asian and Buddhist culture, and those who practice it aim to become aware of the present moment through intentional attention. During the meditation experience, the ability to be open to experiences without judging the thoughts, emotions, and feelings that arise as a result of them can be cultivated [13]. It can be considered that mindfulness is the opposite of “mindlessness” [1].

One construct rooted in this concept of mindfulness, and specifically applied to the parenting context, is *mindful parenting*, which involves the use of mindfulness applied specifically to the parent–child relationship [14]. Positive parenting implies the following: (1) showing affection and support to children, as well as communicating with them and acting as an active agent in stimulation; (2) structuring daily routines; (3) establishing limits, norms, and consequences; and (4) accompanying and getting actively involved in the daily lives of the children [2]. The cultivation of mindfulness supports the exercise of parenting so as to make it a more comprehensive process, favoring satisfaction in the parent–child relationship, which is crucial for the child’s development, socialization, and wellbeing [15]. Parenting requires training in different areas, although it begins with self-awareness and self-care in order to take care of others (in this case, children). The training must be directed to adopting an attitude of non-judgmental acceptance towards the self as a parent, developing self-regulation in the parent–child relationship, and exerting the awareness on the interactions in the framework of parenting [6]. The self-regulation approach is the crucial element required to prevent the situations that arise in this context from being perceived as overwhelming, and to ensure that mindfulness practices favor awareness in order to plan the necessary actions, spending time to balance them with the aim of developing non-automatic responses. Training and learning regarding self-regulation have shown a positive impact on the lives of caregivers and their children, improving their relationships and even some behavioral problems in their children [16].

Duncan et al. [7] propose a model of mindful parenting with five core dimensions necessary for the practice of mindful parenting:

(1) Listening with full attention, which consists of having the intention of hearing beyond the words that are said, paying attention to other aspects that are immersed in them, such as their purpose or their consequences;

(2) Non-judgmental acceptance of both self and child, which implies not making attributions or generating expectations that could interfere in the relationship between parents and children. In addition, this dimension implies not transmitting subconscious perceptions to the children biased by the parents’ wishes. As an alternative, acceptance based on understanding and empathy must be promoted;

(3) Emotional awareness of both self and child, which consists of focusing attention on internal states and being aware of them in order to reduce automatic cognitive processes and to identify emotions. In this way, it is possible to interact with the children in a conscious way and make reflexive decisions that will help to provide a more comprehensive view of the situations;

(4) Self-regulation in the parenting relationship, which is based on reducing negative impulses by adopting a space for reflection that enables the parent(s) to meditate before reacting automatically which, in turn, helps them to respond instead of just reacting;

(5) Compassion for both self and child, which is based on adopting a position of acceptance and empathy and a proactive attitude in the communication with the children to satisfy their needs and provide comfort in the moments when positive affection must be promoted [7].

### 1.3. The Mindfulness-Based Stress Reduction Program

Taking the discipline of mindfulness as a reference, intervention programs have emerged with different objectives depending on the needs detected in the population. Among the most rigorous and scientifically supported programs is the Mindfulness-Based Stress Reduction (MBSR) program, whose main objective is reducing stress levels [17]. According to the theoretical foundations of the program, reduction in stress levels would be a consequence of being aware of the signs of stress when they appear, and its different manifestations in terms of bodily signs, emotions, and thoughts, along with the learning of strategies and tools for stress management [18].

The MBSR program was created in 1979 by Jon Kabat-Zinn, who combined Eastern Buddhist practices and yoga with Western culture, where those practices were unusual [1]. This program was initially designed to be implemented with people suffering from chronic pain and with people who were immersed in clinical contexts. The fundamental reason for choosing this target population was that they had to learn to live with the pain and symptoms that limited their daily lives; thus, learning tools that promote self-regulation, mediated by mindfulness-based meditation, would be beneficial. This aspect helped to modify the subjects’ perceptions of pain, their focus of attention, and their relationships with pain [18].

Research on mindfulness—and specifically on the benefits of MBSR—has grown in the past three decades. Studies have shown that people who use mindfulness-based meditation improve their ability to cope with their problems [19]. There is evidence of significant improvements in stress in general and in its expressions, such as peripheral manifestations and habits related to stress, cardiopulmonary activation, muscle tension, depression, anxiety/fear, emotional irritability, and cognitive disorganization [20]. In addition, participation in the intervention has been shown to improve general mood and, in particular, anxiety, tension, depression, anger, hostility, and fatigue [20,21]. Therefore, these benefits are associated with reductions in stress manifestations, such as decreases in physical and psychosomatic symptoms that affect general health, which generate limitations in daily life. The MBSR program alleviates the suffering associated with the impact of stress on general health, helping participants to cope with their clinical and non-clinical problems, in addition to promoting a change in their perceptions of wellbeing [19].

The wellbeing perceived in parents becomes especially important when they are involved in contexts of psychosocial risk. It has been shown that stress levels and different types of mental health problems reduce the perception of parental role efficacy in raising children, and that the latter are affected by this perception [22]. The practice of conscious parenting may help to increase the parental role efficacy, providing a more accurate view of the reality, and enabling understanding of the implications of their parental actions, by providing psychological flexibility in the field of parenting, which translates into greater conscious and deliberate involvement in parenting responsibilities and tasks [23].

Regarding the benefits to the relationship between parents and children, research indicates that there are changes in parenting components, particularly in terms of the behavioral repertoires that they display and carry out with their children. There is an increase in their motivation and attention towards the parent–child relationship and parenting practices, as well as in their ability to accept their children. Parents have also reported improvements in their self-control and a decrease in reactivity [5,7,16].

Among the changes that are reported by parents, one of the most important skills for the exercise of parenting that helps to improve the relationship between parents and children is emotional intelligence [24]. Emotional intelligence could be defined as the emotional competence to manage emotions, feelings, and thoughts; its objective is to enable individuals to interact with their environment in a rational way in order to promote the capacity to adapt to different contexts and situations and build relationships that are satisfying and promote personal growth [25]. Emotional intelligence seems to function as a mediating variable between mindfulness and self-esteem [26]. This relationship has been corroborated by several studies that have shown that emotional intelligence increases with the practice of mindfulness [27]. Moreover, it has been revealed that these improvements are seen due to participation in the mindfulness-based intervention, since those who benefited from the increase in emotional intelligence were the experimental group and not the control group [24,28]. All of these improvements are seen due to continued mindfulness training, which increases mindfulness levels if the practices are carried out daily [12].

Furthermore, it has been found that the benefits of parental meditation practice are bidirectional and immediately transferable to the children, who are indirectly influenced by the improvement of the parents [14]. For example, in children with developmental delays, some studies have found a decrease in externalizing problems and an increase in attention after their parents’ participation in a mindfulness program [14,16,29]. Parents of children with mental disorders could benefit from this type of program in terms of stress reduction and problem management skills, as it would help them to cope in a more positive way with the challenges and demands associated with parenting children and adolescents with mental health disorders. In addition, it could be beneficial for the wellbeing of the children and adolescents.

### 1.4. Gaps in the Mindfulness-Based Stress Reduction Literature

Despite these advances, and the achievements obtained through the practice of mindfulness, some gaps have been found regarding the implementation and evaluation of intervention programs based on mindfulness in general and, specifically, of the MBSR intervention program. Firstly, one of the current challenges in terms of program evaluation is related to knowing which mechanisms are involved in the changes produced by the intervention—that is, how and why there is a relevant improvement in the quality of life of families after participating in an intervention program [30]. Mindfulness-based programs claim that, through the cultivation of mindful attention and awareness and meditation practices, changes can appear and help individuals to cope with situations that arise in everyday life in a more adaptive way [28]. To advance this field of knowledge, it is necessary to determine which practices are being carried out during the intervention process, and which of them generate changes after the intervention [31].

Another challenge that must be addressed is related to demonstrating the durability of the changes that are achieved through parenting support interventions—that is, whether the changes produced by the intervention remain over time [32]. Considering that parenting support interventions, including mindfulness-based interventions, are based on an experiential methodological approach [31,33], it seems necessary to explore the extent to which the learning obtained through participation in the intervention is applied in everyday life—that is, whether the intervention has really been significant for the participants and, therefore, the acquired strategies can be generalized to other contexts. From a constructivist position on learning, the benefits of an intervention are closely related to the time dedicated to the practices, and require training activities as similar as possible to future contexts of use, as learning depends on the context generated [34,35]. For that reason, MBSR seeks to establish a routine of formal and informal practices in different contexts [36]. To make a habit of meditation practices is a primary goal of MBSR and, to achieve this, it is necessary to begin by carrying out formal practices in the intervention sessions.

Considering the field of parenting specifically, although the MBSR program shows some evidence of effectiveness in the general population [17] and in families in circumstances of vulnerability and/or at psychosocial risk [1], taking into account the characteristics and particular challenges that these families face, it would be interesting to incorporate a more specific parenting component in addition to mindful parenting in this intervention, and to evaluate the effectiveness of this psychoeducational intervention as a parenting support action. Parenting support is defined as actions aimed at improving the wellbeing of families through informative, educational, training, or parental counselling, with a special emphasis on parent–child interactions [37]. Thus, the purpose of these actions is the promotion of resources and competencies in parents so as to optimize the development of children and adolescents, as well as the family’s wellbeing [38,39]. More specifically, parenting support initiatives with a psychoeducational approach are defined as learning/teaching situations, guided by a professional, and aimed at providing parents with opportunities to optimize their parental role through cognitive, emotional, and behavioral changes [38]. In addition, psychoeducational interventions in the family domain usually incorporate a community intervention component, strengthening informal support networks [40].

Therefore, in MBSR—understood as a psychoeducational parenting support program—the parenting element could be incorporated as a specific component and as a transversal element in the intervention [2]. Firstly, the contents of the intervention are aimed at providing opportunities for emotional, cognitive, and behavioral changes as well as the activation of internal resources. Secondly, the use of informal home practices to apply the contents learned during the sessions puts the focus on their parenting role, guaranteeing that the training in situations is as similar as possible to the future context of use. In concert with the use of community intervention components in psychoeducational approaches, the use of a group format would enable the sharing of parenting experiences and the re-elaboration of personal perspectives via discussion with other parents to provide alternative models [34]. In addition, sharing experiences with other caregivers who experience similar situations leads to feeling integrated and understood in the group [41]. Finally, incorporating specific parenting sessions could promote more comprehensive learning, allowing specific support needs and demands that these parents face to be addressed, and putting more emphasis on certain parental competencies and strategies that are particularly relevant for these parents [42]. Some of the demands that could be addressed within these positive parenting sessions include the knowledge of the essential tools and resources to be able to adequately understand the health problems of their children, along with coping strategies to deal with these situations. As being immersed in these circumstances causes feelings of confusion, disbelief, anxiety, and fear [4], an intervention program that seeks to address these feelings by providing strategies and tools that help to manage these situations could be useful for them.

Moreover, we do not have enough information about the effectiveness of this intervention for families in vulnerable situations, such as parents with children with mental health disorders. It is necessary to provide evidence for the effectiveness of the MBSR with a positive parenting component, and to determine whether after their participation the parents managed to mitigate the negative consequences for parenting by taking into account their family circumstances. Knowing the effectiveness of this intervention for this type of population is of particular interest, considering the high levels of stress in these families [2], and that stress reduction is a central element of the MBSR, among other mindfulness-based modalities [13,18].

Finally, research on the implementation and effectiveness of mindfulness-based programs has predominantly involved the use of quantitative methodologies [43]. The combination of quantitative and qualitative techniques and strategies would enrich the evaluation of mindfulness-based programs, allowing consideration of the integral views of the participants, and providing a more complete representation of the complexities behind the learning, integration of practices in daily life, and the implementation process [44]. This will help to provide a better understanding of how the program works in the target population, and to enable it to be adapted and improved so that it is as beneficial as possible [36,42]. Along with this, satisfaction evaluations should be included regarding the core components of the program, in order to obtain the views of the participants so as to accommodate their needs and demands and optimize the process [45,46].

### 1.5. Research Objectives

Taking as reference all of the aspects mentioned above, the aim of this protocol is to incorporate the positive parenting component in the original MBSR program, and to carry out an effectiveness evaluation with parents of children with mental disorders, along with analyzing the participants’ perceptions of change in order to ascertain how the intervention influences their daily lives and their relationships with their children. To sum up, the MBSR evaluation protocol for parents of children with mental disorders who participate in this program has the following objectives: (1) to systematize the incorporation of a parenting component in the MBSR program; (2) to analyze the effectiveness of the MBSR program with the added parenting component (named MBSR-P) in parents of children with mental health problems; and (3) to examine the mechanisms of change present in the MBSR-P, as well as the application of acquired strategies to daily life.

## 2. Materials and Methods

### 2.1. Study Design

This study follows a longitudinal, quasi-experimental design with two parallel groups (intervention and control group) and three evaluation moments (pre-test, post-test, and follow-up 6 months after the end of the intervention), with a mixed methodological strategy combining quantitative and qualitative information. The study population will be the parents and caregivers of children who benefit from specialized mental health services in the region where the study will take place.

### 2.2. Measures

The participants will fill out a battery of instruments aimed at collecting information on their sociodemographic profiles, assessing the effectiveness of the intervention, and examining the changes perceived by those participating in the mindfulness-based psychoeducational program. All of the instruments will be administered in the native language of the participants (Spanish). For the questionnaires used to evaluate the effectiveness of the program, the validated Spanish version of each of them will be used. The ad hoc questionnaires have been developed by the research team, and are available in Appendix A.

#### 2.2.1. Sociodemographic Profile Measures

Before starting the intervention, a questionnaire designed ad hoc will be administered in order to obtain information on the sociodemographic profiles of the families (Table A1). Specifically, questions regarding age, sex, educational level, professional situation, and job stability will be included, in addition to the age, sex, and diagnostic category of the child. Regarding mindfulness, we will ask whether the participants have had previous experience in participating in mindfulness-based interventions, and whether they take part in any relaxing activities. Regarding the families, information will be collected on the type of family, its size, and its stability. The questions were selected considering basic sample profile information such as age and gender. Considering that parents are the target population, questions concerning the family context will be included. Finally, questions about elements that could have an influence on the impact of the intervention—such as the previous experience with and use of alternative stress management strategies—will also be incorporated. Part of the research team presented a proposal following the format of other sociodemographic questionnaires previously used by this team. Afterwards, a senior researcher with ample experience in program evaluation and a practitioner with experience in mindfulness-based interventions reviewed the questions and approved them.

At the same time, the Family Affluence Scale (FAS II) [47]—a scale with four items developed to measure the family socioeconomic level—will be administered. This instrument has obtained good criterion validity data, with a kappa coefficient of agreement of 0.57. Metric scores will be calculated through totals, and the scores will be grouped into low, medium, and high categories. For the grouping of the categories of low, medium, and high SES, the national standards that have been established through multinational analyses will be considered [48].

#### 2.2.2. Effectiveness Measures

The effectiveness measures will be collected at pre-test, post-test, and follow-up. These measures are described as follows:

Stress. The first measure is related to the levels of perceived stress during the past month, which will be evaluated through the Perceived Stress Scale (PSS) [49,50]. This questionnaire contains 14 items (e.g., “In the last month, how often have you felt nervous or stressed?”). The scale of Likert responses ranges from 0 = *never* to 4 = *very often*, where 4 means the highest level of perceived stress. The instrument score is obtained from the mean of the scores, whose values range between 0 and 4. Research on the reliability and validity of this instrument shows internal consistency and validity, with a reliability value of α = 0.81.

Mindfulness. The second measure will be the evaluation of the levels of mindfulness and awareness in the present moment over a short period of time through the Mindfulness Attention Awareness Scale (MAAS) [51,52]. This instrument is composed of 15 items (e.g., “I do things quickly and hastily without paying much attention to what I am doing”), and uses a 6-point Likert scale, between 1 = *almost always* and 6 = *almost never*, where higher scores indicate a higher degree of mindfulness. The instrument score is obtained by calculating the mean of the scores, whose values range between 1 and 6. Research on the reliability and validity of this instrument has shown high internal consistency, with a Cronbach’s alpha of α = 0.88, and with significant values of criterion and discriminant validity.

Emotional Intelligence. The third measure is related to emotional intelligence, which will be assessed using the Emotional Quotient Inventory Short Form (EQi) [53,54,55]. This scale has 35 items (e.g., “When facing a problem, the first thing I do is stop and think”), which are divided into 4 subscales: intrapersonal intelligence, interpersonal intelligence, adaptability, and stress management. The response to the items is on a Likert-type scale, ranging from 1 = *very rarely true in me* to 5 = *very frequently true in me*. The score of the instrument is obtained from the means of the items belonging to each subscale, whose values range between 1 and 5. The reliability data of the instrument are acceptable in all subscales: 0.73 in the intrapersonal dimension, 0.78 in the interpersonal dimension, and 0.70 and 0.75 for adaptability and stress management, respectively.

General Health. The fourth measure will consider the general aspects of the symptoms regarding general health in recent weeks, which will be measured with the General Health Questionnaire (GHQ) [56,57]. This questionnaire consists of 28 items (e.g., “Have you felt perfectly well and in good shape?”) grouped into 4 subscales of 7 items each: somatic symptoms, anxiety and insomnia, social dysfunction, and severe depression. The responses range from 0 = *not at all* to 3 = *much more than usual*, and the instrument score is obtained via the means of the items belonging to each subscale, whose values range between 0 and 3. This instrument has shown high test–retest reliability (*r* = 0.90) and satisfactory data regarding predictive validity.

Parental Role. The fifth measure refers to the perception of efficacy of the parental role, which will be evaluated with the “Me as a Parent” (MaaP) scale [58]. This instrument has 16 items (e.g., “I trust myself as a parent”), which measure 4 factors of 4 items each: self-efficacy, personal agency, self-reliance, and self-management. The items have Likert-type responses that range from 1 = *strongly disagree* to 5 = *strongly agree*, where the highest scores reflect higher levels in each construct; thus, in order for the personal agency factor to have the same measure, the responses to the items must be reversed. Mean scores can range from 1 to 5 on the general scale and on each of the subscales. The data on the reliability of the global scale show good internal consistency (α = 0.85), and the data referring to facial, factorial, and convergent validity are also satisfactory. Regarding the reliability of the subscales, they all have α values above 0.60.

#### 2.2.3. Perceived Impact by the Participants and Implementation Analysis of the Program

In order to evaluate the perceived impact by the participants and the implementation of the program, halfway through the intervention, in the sixth session, the participants will fill out a questionnaire composed of nine open questions designed ad hoc to obtain feedback from the participants on the intervention process (Table A2). The questions will refer to the expectations met up to that moment, the learning obtained and its integration into daily life, the changes perceived, the implementation of the practices, the difficulties encountered, and the methodology used (e.g., “Do you notice changes in the physical body/mind as a result of these practices?”). These questions were based on the mid-program questions proposed in the original MBSR; the authors then adapted them to the program and intervention context, incorporating questions considered relevant by the authors—who have experience in program evaluation—to adequately fit the characteristics of this intervention. The open questions were developed so that they were relevant, clear, concise, and specific, without overlapping with others. Finally, a practitioner with experience in mindfulness interventions reviewed the questions and adapted their language and formulation [59].

Moreover, the professional who leads the group will fill out a field diary after each intervention session, where information will be collected about the implementation of the program, as well as the group dynamics and the implementation of informal practices at home (Table A3). The questions will have both open and closed answers (e.g., “Any notable issues in the session? Strengths of the program, aspects of the program that have not worked well, or other observations about the course of the session”).

A discussion group will be carried out at post-test (in the last session), in which the following questions will be presented: (a) “Are you satisfied with the program? What has it given you in a personal and professional context?”; (b) “What are the elements or components of the program that have helped you the most? Why?”; (c) “What do you think about the intervention being carried out in a group? Has it helped/contributed or not? In what way?”; (d) “What moments in the intervention would you consider to be the turning point for you?”; and (e) “What do you think could be improved about the program in the future?”.

Finally, with the aim of evaluating the application of the learning to daily life achieved through participation in the intervention, the participants will fill out a questionnaire 6 months after the end of the intervention, in the follow-up phase (Table A4). This questionnaire will be made up of both of open and closed questions (e.g., “In what aspects have you included mindfulness into your daily life? What has helped you to do so?”).

#### 2.2.4. Client Satisfaction

This evaluation will be carried out using the Client Satisfaction Questionnaire (CSQ) [60,61], which is composed of eight items (e.g., “How would you rate the quality of the service you have received?”), each of them with a 4-point Likert-type scale. The weighted score ranges between 1 and 4, with the highest scores indicating the highest satisfaction. Research on the psychometric properties of the instrument indicates good internal consistency (α = 0.93) and concurrent validity [60].

### 2.3. Study Setting

The implementation of the MBSR-P will be carried out in the specialized mental health facilities for children and adolescents of the region where the study will take place. In this region, mental health services offer specialized programs of mental health care for the population that needs it, including children and adolescents. The portfolio of care programs in this resource is made up of outpatient care, day programs, and full hospitalization [62,63].

In these specialized services, the patients regularly visit hospital facilities and dedicate a large amount of time to health care. This immersion sometimes causes personal wear and stress, especially for the patients’ relatives, and specifically for the main caregivers (mostly parents), who must face added and unknown responsibilities. For this reason, it is of great relevance not only to attend to the patients, but also to provide professional attention to the people who accompany them, in order to minimize the negative impact on their lives and allow them to reconcile the latter in a positive way.

### 2.4. Participants

#### 2.4.1. Characteristics

The study sample will be made up of caregivers of children with a mental disorder who receive attention from specialized mental health services.

In this context, the professionals who work in that specific hospital unit have observed the overload of caregivers due to the high responsibility involved in taking care of their children in their particular circumstances, thereby corroborating the need to provide care for caregivers [4].

The care proposal from the mindfulness program emphasizes taking care of oneself in order to be able to take care of others in an effective way, through the use of strategies that help to manage difficult day-to-day situations, which are sometimes a source of stress for parents. Emphasis is placed on the relationship with oneself and on the acceptance of one’s own feelings, so as to then focus on communication and conscious relationships with the person(s) cared for.

In general, this protocol seeks to provide complementary care for informal caregivers who seek to contact other caregivers who live the same experience, with the aim of being able to share it and learning other ways to cope with their circumstances and to activate internal resources as a means to overcome stress, pain, and daily challenges. Likewise, it is intended to give parents the opportunity to optimize their parental performance.

#### 2.4.2. Recruitment, Inclusion, and Exclusion Criteria

For the selection of the sample, a non-probabilistic sampling will be carried out by accessibility to the target population. The participants will be informed of the intervention through posters that will be placed in the waiting room of the specialized mental health services for children and adolescents, and through the health care professionals who work in that service and who care for the families. The professionals will inform the caregivers who, in their opinion, could benefit from participation, referring them to the program, thus promoting the participation of parents with higher support needs, and complementing the previous recruitment method. After being duly informed and knowing the most relevant points of the program, the caregivers who are interested in carrying out the intervention will voluntarily fill out a document with their identification data and deliver it to the hospital administration to record their registration. The professional who will implement the program will contact the interested people in order of request, prioritizing parents who are referred to the program by the mental health professionals, and will invite them to an informative meeting about the program to provide a first contact and explain what the program would consist of and its purpose.

The inclusion criteria for participation are: (1) being a caregiver for a child with a mental health disorder and receiving care from the specialized mental health services for children and adolescents; (2) not having previously participated in the same MBSR group; (3) not experiencing a major life crisis; (4) not having suicidal ideas at the time of intervention; and (5) not showing signs of addiction to toxic substances.

Since randomization of the groups will not be possible, the control group will be made up of subjects comparable to the intervention group in terms of their sociodemographic profiles, via a matching strategy aimed at guaranteeing equivalence between groups [46]. We will consider three different variables to match the control group: (1) age of the child, divided into two categories (children or adolescents); (2) diagnostic category of the child (differentiating between externalizing disorders, internalizing disorders, or others); and (3) sex of the parent. Parents enlisted in the waiting list to participate in the intervention will be used as the control group in order to guarantee recruitment and retention.

### 2.5. Procedure

#### 2.5.1. MBSR-P and Implementation

For this research, the MBSR program created by Jon-Kabat Zinn et al. in 1979 at the University of Massachusetts will be applied, with some adaptations. The MBSR is a psychoeducational intervention program that has mindfulness as its backbone, and whose objective is to provide strategies for the management and awareness of stress itself. It is an instructive and multimodal intervention that is carried out in a systematic and structured manner [59].

The original MBSR program lasts 8 weeks, with weekly sessions of 2.50 h, along with an intensive day of 6 h and daily homework of 45 min, with the aim of integrating the practices and generalizing them to daily life. Intervention groups typically range from 10 to 40 participants [13,19].

As mentioned above, taking into account the implementation context, the target population, and their circumstances, a series of adaptations will be made to the original MBSR program, as indicated [64]. The adaptations will be aimed at the timing of the intervention, the informal practices, and the contents. Regarding the timing of both the formal sessions and the recommended informal practices, the required duration will be reduced. The formal MBSR-P sessions will last 1.50 h (except for the informative session, which will last 2 h, and the intensive session, which will last 3 h), and the home practices will last 30 min. Concerning the contents’ adaptation, two specific sessions dedicated to promoting positive parenting will be incorporated. Therefore, the complete program will consist of 11 sessions: a first informative session, 8 weekly sessions based on MBSR, and 2 positive parenting sessions aimed at promoting parenting adapted to the children’s needs as well as awareness of their own parenting practices. The program includes home practices each week. The home practices will be introduced in the last part of each session, and the necessary materials to be able to carry them out will be sent via email. The program is expected to have a total duration of 3 months.

Thus, the rationale behind how the program is organized is that throughout the eight MBSR-based sessions parents will work on activating their internal resources which, in turn, will help them to cope with the challenges they face in their daily lives in their parenting roles. In addition, the home practices and the group dynamics will allow parents to implement what they have learned in the different sessions in their family lives and, particularly, in their parental roles. The last two specific positive parenting sessions will have a complementary role in terms of reinforcing the application of the strategies and resources learned in the previous sessions in their parenting roles, and will provide the parents with additional strategies to cope with their situations and attend to specific needs, such as communication difficulties.

In all sessions, a specific structure will be followed: the session will begin with a group meditation; later, time will be dedicated to sharing the home practices from the previous week, and individual experiences will be presented in the large group; then, the contents planned for the session will be introduced, and will be practiced through meditations and other exercises, which will also be done in small groups; finally, in the last part of the session, the home practices planned for that week will be presented. The sessions will finish with a group meditation. Table 1 presents the detailed contents of each session.

Regarding the materials required to carry out the practices in the formal sessions, it will be necessary to have a large space equipped with mats and chairs for each participant, so that they can perform every type of meditation comfortably. The necessary materials to carry out the home practices (audios, videos, readings, schemes) will be provided by the professional responsible for the group.

#### 2.5.2. Process Evaluation

In the first meeting, the participants will be informed about how the program will be carried out, the general objectives of the program, their participation in it, the content of the sessions, the importance of their engagement with all of the sessions, the materials that will be needed, and the guidelines to follow throughout the process, such as carrying out activities at home. Once the information has been received and any doubts clarified, if they agree with all of the points, the participants will sign an informed consent form in accordance with the Declaration of Helsinki [65], in which they will be informed that the discussion group will be audio recorded, and that confidentiality will be ensured for all participants. The informed consent will also notify the participants that they can leave the intervention at any time without this decision having any consequences. There will be no financial or other incentives for participation.

In this first session, the pre-test data from the battery of self-administered questionnaires will be collected (including both the sociodemographic profiles and the effectiveness measures) from the intervention group. In the intermediate phase of the intervention (sixth session), the participants will fill out the questionnaire to evaluate the feedback of the intervention process up until that moment. At the end of the intervention, the post-test data from the battery of effectiveness and satisfaction self-administered questionnaires will be collected, and the discussion group will be held. Furthermore, during the intervention process, after each session, the professional in charge of the implementation will fill out the field diary corresponding to each session once the session is over. Over a period of 6 months after the end of the intervention, the follow-up will be carried out, in which the battery of instruments referring to the effectiveness of the intervention will be administered to the participants, in addition to the follow-up questionnaire aimed at evaluating the application to daily life of the learning achieved through participation in the intervention.

The control group will fill out the pre- and post-test batteries of questionnaires (sociodemographic profiles and effectiveness measures) in a comparable time frame to the intervention group. This data collection will be carried out under the same terms, conditions, and time periods as in the intervention group. Two people will be involved in the data collection, both with prior training in the collection and analysis of both quantitative and qualitative data. During the completion of the questionnaires, any doubts that may arise will be clarified individually to eliminate bias.

In relation to the confidentiality of the data, each person will be assigned identifiers to archive them, and secure cloud content management software will be used to save the data.

**Figure 1:**
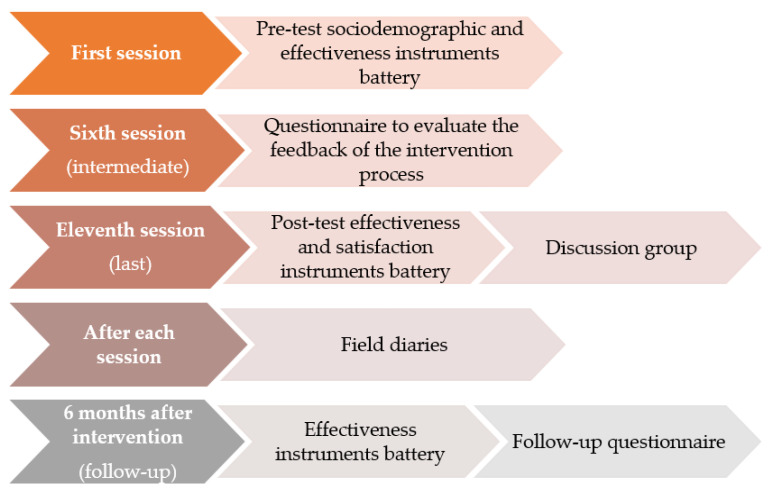
Evaluation process of the MBSR-P intervention program.

Figure 1 shows the evaluation process that will be carried out—specifically, what information will be collected at each moment.

#### 2.5.3. Sample Size

To calculate the sample size necessary to achieve adequate statistical power in the evaluation of the program, the G*Power 3.1 program was used [66]. Two calculations were made for the sample size. The first calculation that was carried out for the pre-test and post-test analyses was made a priori for ANOVA analysis of repeated measures within–between interaction, with a mean Cohen effect size *f* = 0.25 [67] and α = 0.05, two groups with two temporal measures, correlation between the measures of 0.50, and sphericity of 1. With these assumptions, to achieve the acceptable minimum Cohen effect size of 0.80 [68] and obtain a statistical power of 0.80, the sample should consist of at least 34 participants. Although regarding the size of the control group, following a common knowledge rule in program evaluation, the control group should be composed of a minimum of (*n*/2) + 1—where *n* is the sample size of the group of participants—in this study, a minimum of 20 participants is established for the control group, with the objective of reaching the recommended size for the parametric tests [69].

The second sample calculation that was carried out for the follow-up analysis of the intervention group was performed a priori for the ANOVA analysis of repeated measures within factors, with a mean Cohen effect size *f* = 0.25 [67] and α = 0.05, a group with three temporal measures, correlation between the measures of 0.50, and sphericity of 1. With these assumptions, to achieve the acceptable minimum Cohen effect size of 0.80 [68] and obtain a statistical power of 0.80, the sample should be made up of at least 28 participants.

Among the analyses that were conducted, the most restrictive was the first calculation referring to pre-test and post-test measures of the intervention group and the control group; thus, we assume that the study sample must consist of at least 34 participants for the intervention group and 20 participants for the control group.

### 2.6. Data Analysis

#### 2.6.1. Quantitative Data

The analysis of the quantitative data will be carried out using the statistical program SPSS v-21 (IBM Corp., Armonk, NY, USA) [70]. Firstly, missing data at the level of the Likert scale questionnaires will be studied. Little’s MCAR test will be used to verify whether missing data per questionnaire are below 10%, which will mean that they are randomly distributed. If this assumption is met, it will also be verified whether missing data per item have a percentage below 5% and, if so, the data will be imputed through the SEM procedure using the expectation maximization (EM) algorithm from SPSS. For those cases with missing data at the questionnaire level, the cases will be discarded [71].

The distribution of the values will also be evaluated through asymmetry and kurtosis, and the presence of extreme cases in the variables studied will be examined by analyzing the interquartile ranges through stem and leaf graphs [72].

Moreover, other statistical assumptions for the parametric tests will be checked and reported (i.e., sphericity, normality, linearity, homogeneity, and absence of multicollinearity and singularity) [73]. If the sphericity assumption is not fulfilled, the *F*-test will be adjusted as an alternative for analysis with the Greenhouse–Geisser index, reducing the degrees of freedom associated with the *F* statistic of the ANOVA as a function of the corrective factor ε [74]. If normality violation occurs, parsimony transformations will be applied to correct the non-normal distributions [75]. If multicollinearity applies, composited factors will be computed for those involved variables.

The sociodemographic variables will be used to describe the samples, to control for them in the main analyses, and to achieve equivalence between the characteristics of the control group and those of the intervention group. Both descriptive and correlational analyses will be performed as preliminary analyses.

The effectiveness of the MBSR-P intervention will be examined by performing two-way repeated measures ANOVA for each effectiveness dimension studied, with the group (0 = *control*, 1 = *intervention*) as the factor. Interaction effects will be examined. *R*^2^ will be computed as the effect size index, and will be evaluated according to the conventional levels of 0.01, 0.06, and 0.14 for the small, medium, and large effect size levels, respectively [67]. The corrected *R*^2^ will be calculated taking into account the within- and between-subjects errors [75]. In the event that the assumptions for these analyses are not fulfilled (e.g., if normality is violated and not able to be fixed by variable transformations), Wilcoxon’s non-parametric tests will be used as an alternative [74]. Mean and standard deviation will be provided for participants’ satisfaction, and for the implementation of information registered in the field diaries.

**Figure 2:**
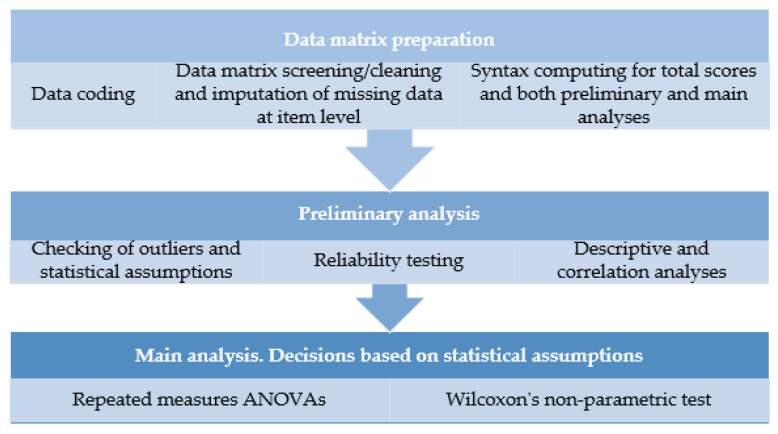
Steps for quantitative data analysis.

Figure 2 shows the steps that will be followed for quantitative data analysis.

#### 2.6.2. Qualitative Data

For the management and coding of qualitative data, the qualitative analysis software NVIVO (QRS, Burlington, MA, USA) will be used [76]. With the information that the narratives of the participants will reveal, through the qualitative methodology, two types of analyses will be carried out depending on the information collected. On the one hand, an enumerative content analysis will be performed on the questionnaire of the intermediate phase of the intervention and on the follow-up questionnaire (Grbich, 2013, cit. in [77]). On the other hand, an inductive thematic analysis will be carried out without predetermined theoretical assumptions of the qualitative data from verbatim transcripts of the audio recordings from the discussion group [78]. The intermediate questionnaires will support the implementation analysis, and will feed the systematization of the intervention. The discussion groups will support the analysis of the mechanisms of change. Finally, the follow-up questionnaires will be useful for the examination of the transferability of the acquired practices to daily life.

The analyses will be conducted in parallel by two researchers with experience in qualitative methodology, along with an external supervisor. First, a code book will be built for each instrument following the recommendations of Braun and Clarke [78]: (1) to familiarize the researchers with the data, and (2) to generate initial codes. Additionally, for the thematic analysis, the recommendations of Braun and Clarke [78] corresponding to the study of the topics will be followed: (3) to search for themes; (4) to review the themes; (5) to define and name the themes; and (6) to write up the results.

Once the category systems are created, the researchers will code separately and, subsequently, meet to discuss the categories and ascertain the degree of inter-observer reliability, which will be calculated by dividing the total number of agreements in the coding of all categories by the total number of agreements and disagreements in all categories [79]. Moreover, for the enumerative content analysis, the percentages of participants that have mentioned each category in their narrative will be calculated in order to obtain comparative data on which elements of the intervention are more frequently mentioned, and the areas in which the program has a greater impact on their daily lives. For the representation of these data, comparative graphs of the percentages of participants in each category will be made, and word clouds of the most common narratives in the categories will be provided. The word clouds will be created to identify the most frequent concepts that are talked about in the focus groups, organized by the previously identified categories and in a visual manner. To do so, a selection of the 50 most frequent words will be carried out, using as an inclusion criterion the length of the word (more than 3 letters). Secondly, words with the same root (e.g., parent and parenting) will be grouped under the same concept (e.g., “parenting”). Finally, we will eliminate linking and non-content words such as “between” or “element”. For the inductive thematic analysis, the two researchers will go back and forth through the data separately so as to identify possible themes. Later on, the proposed themes will be shared, and the researchers will discuss and not only agree on a set of themes, but also identify higher order themes and subthemes, define them in depth, and select quotes that represent those themes and subthemes.

To reach an adequate sample size that will enable us to carry out this content and thematic analysis using a representative sample, we will use the data saturation criteria. Taking into consideration the parameters proposed by Hennink et al. to estimate the sample size in focus groups, we will carry out between five and six focus groups so as to achieve both code and meaning saturation [80]. Code saturation is defined as the moment when no additional codes appear in the data, and meaning saturation is defined in terms of whether the qualitative data provide further information or insight on the codes [80].

**Figure 3:**
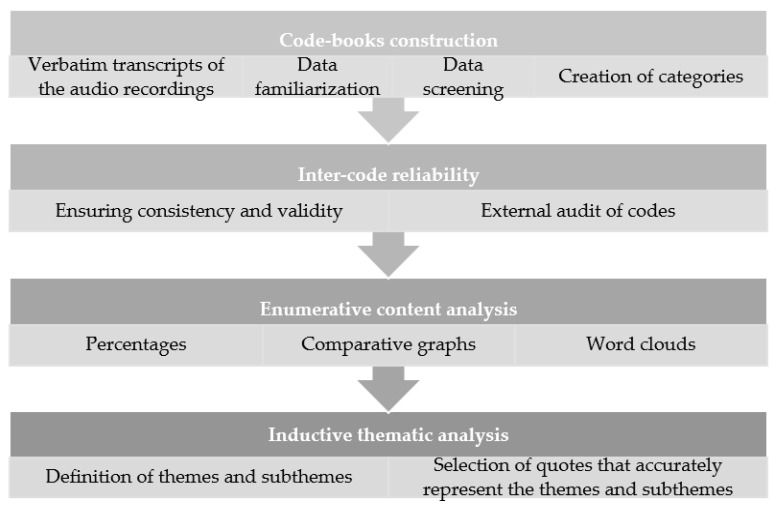
Steps for qualitative data analysis.

Figure 3 shows the steps that will be followed for qualitative data analysis.

## 3. Discussion

The practice of meditation and mindfulness has become widespread throughout the world due to the benefits that have been reported from it [19]. MBSR is one of the mindfulness-based interventions with the most evidence, and this study aims to expand a solid basis for the rigorous evaluation of MBSR with an added parenting component (MBSR-P) for families with children with mental health problems.

### 3.1. Expected Results

The MBSR-P program is a psychoeducational and multimodal intervention adapted from Jon-Kabat Zinn’s original MBSR [13,18], created for caregivers of children and adolescents with mental disorders. Although there is some evidence of effectiveness of the original MBSR program, this proposal seeks to optimally adjust the intervention program to the characteristics and circumstances of caregivers of children and adolescents with mental disorders. For this purpose, positive parenting contents will be incorporated throughout the intervention, two specific positive parenting sessions will be added, and the timing of the sessions and home practices will be modified. Thus, we expect the systematization of the intervention to guarantee the tailoring of the program to the specific needs and demands of this population [81]. The adaptation of the timing of the sessions and home practices take into consideration the already busy schedules of parents of children and adolescents with mental health problems [3,4]. The integration of the intervention in the mental health services considers their use of different services. We also expect to satisfy the demands of parents for both self-nourishing/self-care time and an improvement of parental competencies by incorporating positive parenting contents in a personal development psychoeducational intervention [3,4]. Moreover, with the systematization of the intervention, we also expect to maintain a high level of fidelity in the implementation of the intervention which, in turn, would guarantee the quality of the intervention, facilitating the acquisition of the intended objectives as well as their transferability to the participants’ daily lives [82].

The mixed-methods approach used in this study will help to provide different perspectives and complementary data of a different nature in order to obtain more complete information and understanding of how the program has an impact on different areas of parents’ lives, and how the learning obtained in the intervention context is integrated in the personal and family environment. It will also enable the more in-depth analysis of the implementation process and mechanisms of change, identifying strengths and weaknesses of the program [44].

Moreover, this research seeks to provide evidence of the effectiveness of the MBSR-P program in improving the quality of life of caregivers of children and adolescents with mental disorders, and alleviating negative symptoms through the practice of mindfulness [20]. Taking as a reference the existing literature, we expect changes in areas related to stress, mindfulness, emotional intelligence, general health, and the participants’ perceptions of efficacy in their parental roles [12,21,23,24]. As mentioned in the introduction, with the cross-cutting approach of self-regulation, it is expected that participation in the MBSR-P program will help parents of children with mental disorders to face the responsibility of caring for their children, identifying stressful situations, becoming aware of them, and improving their methods of dealing with them, displaying cognitive and behavioral patterns that are in line with the problem that arises [15]. In addition, with the contribution and use of tools and strategies, the intention is that once parents learn to positively face complicated day-to-day situations, the relationship between parents and children and the family environment are improved, increasing both individual and family satisfaction [7,16]. The analysis of the MBSR-P program will not be limited to its effectiveness; by examining whether the impact of the intervention is maintained in the mid-term and testing the application of the learned strategies in daily life, we will be able to prove whether the intervention has made a real difference in participants’ lives. Although there are no studies to our knowledge on the mid-term effects of MBSR interventions on parents with children with mental health issues, taking as a reference follow-up studies with other populations, we expect the changes in the evaluated dimensions to be maintained in the mid-term [5,83,84]. In relation to the practices learned during training, we expect meditation practices (e.g., body scan and breathing techniques) to be the ones more easily transferred to daily life in the mid-term, as they are less time consuming, do not require additional materials, and can be carried out in any setting [36].

Moreover, by analyzing the narratives of the parents after the intervention, we expect to gain insight about the mechanisms involved in their processes of change during the intervention. First, from the mindfulness-based intervention approach, and based on the existing theoretical and empirical evidence on mediators, we expect non-judgmental mindful attention, self-nourishing attention, awareness of their parental roles, awareness of their own negative patterns of communications and thoughts, and the reduction in reactivity to be identified as the pillar elements that have prompted the changes associated with their participation in the program [6,85,86,87]. Additionally, considering the relevance of home practices for the intervention outcomes, we expect the continuous use of the techniques and strategies learned throughout the program to be considered as an important element for the maintenance of changes during and after the intervention [88]. Second, from the experiential methodology employed in the MBSR-P program, we expect that participants will highlight the relevance of focusing on their own experiences to build up their learning by remodeling their beliefs, knowledge, and abilities [41]. Third, the group format is expected to favor social learning, and to be reported by the participants in terms of modeling, cognitive insight, emotional expression, or interpersonal learning [89].

### 3.2. Limitations

Finally, this study has some limitations that ought to be stated. First, the limits inherent to a real-context evaluation prevent the use of a randomized controlled trial design that would improve the rigorousness of the study approach [90]. The comparison of the MBSR-P program with the original MBSR to test the specific added value of the positive parenting component is another challenge that the authors hope to test in near-future research with a population with common characteristics in terms of the needs and demands that they face, such as parents of children and adolescents with other difficulties, such as physical health conditions [91,92]. Moreover, although giving voice to the participants is a valuable approach for understanding key intervention aspects involved in the process of change, direct testing of theoretical models is needed in order to fully map the mechanisms of change involved in the intervention [93]. This challenge could be addressed in future research benefiting from the results obtained in the current study. Despite said limitations, we think this study has relevant practical implications, as described below.

### 3.3. Practical Implications and Dissemination Plan

Mental health services are usually focused on direct interventions with children and adolescents, while parents and caregivers are often companions or helpers throughout. These services sometimes target the family as a whole; however, the wellbeing of parents as individuals and support in their parental roles are often overlooked. In addition, parents’ perceptions of their lives revolving predominantly around the caregiving of their child—not having personal time for leisure activities or self-care—often lead to an increase in stress levels and feelings of dissatisfaction with life in general [2].

Studies have shown the importance of the wellbeing of caregivers in order for them to be able to cope with the task of caring for children with mental health problems for both the prognosis of the child and the improvement of intrafamily relationships [12]. With this protocol, we seek to support a currently neglected sector, valuing the effort, perseverance, and dedication of the caregivers, which means highlighting the great relevance of their task, and the additional demands that these parents face. To adequately perform their parental responsibilities, caregivers need to have informal and formal support systems so that they can meet the different demands that they face and prevent negative interference with their daily lives and the childcare process [94].

Detailed manualization of intervention programs is crucial in program implementation and evaluation research. Manualization of the program is an important step towards guaranteeing the fidelity of the theoretical and methodological principles underpinning the intervention program and the implementation process, as well as its replicability and future dissemination among other practitioners [45,46,95]. The systematization of the evaluation protocol will guarantee a rigorous and multimodal evaluation process where adequate evaluation strategies are used. In addition, it will help to test and ensure the fidelity of the implementation process, while being flexible with the possibility of changes to adapt the intervention to the needs and demands of each group of parents [82]. This systematization will also help researchers to study and test the MBSR-P using similar methodological processes, enabling data comparability in order to provide further scientific evidence [45,46]. Therefore, the systematization of the evaluation process will enhance the intervention and implementation process, as well as facilitating the decision making concerning the allocation of public resources to support families of children and adolescents with mental health disorders, prioritizing evidence-based interventions [96,97,98].

The inclusion of measures that target mechanisms of change will promote further understanding of how the changes that take place during the intervention are related to the key components of the MBSR-P. These data will also help to improve the implementation process by identifying those practices and moments in the intervention that are key to reaching the program’s objectives [30,31]. Thus, these measures will help to further adapt the intervention to the characteristics and demands of the targeted population [81]. Transferability of the practices to daily life situations is another key component of the program. This part of the evaluation protocol will be crucial to identify facilitators and challenges in the application of the techniques and strategies to the daily lives of parents of children and adolescents with mental health disorders, as well as the influence of daily practices in the maintenance of changes in the short and medium term [93].

Regarding the dissemination plans, the results will be disseminated in different fields; one of them will be among professionals working with children and adolescents with mental health disorders, so that they are aware of the existence of the resource of the MBSR-P program to provide support to the parents of children and adolescents being treated in different services. For this purpose, information about the program and the results of the research will be incorporated into the internal coordination structure of the health service. In addition, brief online presentations will be made for practitioners from different fields who could also find this program helpful for their services. Moreover, this protocol and the results of the research will be disseminated in congresses that target practitioners. In the academic field, the data will be disseminated by publication in international high-impact journals in the field and making communications in relevant congresses.

## 4. Conclusions

This study is aimed at manualizing and systematizing the application of the MBSR-P intervention, as well as rigorously studying both its processes and its effects, with the purpose of being able to generalize its use and provide knowledge about its effectiveness, contributing to the design of a new parental support proposal for groups with specific needs, such as parents of children and adolescents with mental disorders.

The careful study of the program will provide a breakthrough in its understanding via analysis of the aspects involved in the processes of change through the experience of the participants in the meditation practices, which will help not only to provide evidence, but also to identify the key components of the interventions based on mindfulness and its implication in the changes.

Likewise, the analysis of the application of meditation practices to daily life can help to identify the challenges to achieving its transferability. These challenges and limitations may be related to both the participants and the components of the program, as well as its implementation. Addressing these limitations will give us clues about the keys to guaranteeing transferability, which has important implications at both the research and intervention levels.

## Figures and Tables

**Table 1:** Sessions and contents of the MBSR-P.

Session	Contents
1	Informative session: Presentations, brief explanation of the program, guidelines to follow during the program, group norms, and recommendations to obtain the maximum benefit. Pre-test sociodemographic and effectiveness instruments battery;
2	Introduction to mindfulness: Theoretical and conceptual bases of the program, application of self-regulation skills, and introductory breathing practices. Theoretical explanation of the seven bases of mindfulness, such as nonjudgement, trusting, and beginner’s mind. Some of the practices will include the raising exercise to refine the senses and be aware of how one perceives one’s experiences through one’s senses. Home practices will be 10 min meditations focusing on breathing;
3	Finding your anchor: Meditation centered on breathing, and the foundation of the mindfulness triangle of thoughts, emotions, and sensations. Introduction of some inspirational readings, so as to be aware of the emotions, thoughts and sensations that emerge during those readings. Home practices will be 2 min breathing exercises and inspirational readings to observe the triangle previously described;
4	Corporal scanning: Conscious stretching exercises. Body scan, guiding practice so as to be able to become aware of the sensations that are emerging through attention to breathing. Sequenced aware movements will also be introduced. Home practices will be 10 min meditations and a calendar of positive events, where participants will write down certain positive events that have happened throughout the week in a systematic way, following certain directions;
5	Connecting with stress: Reflection on stressful experiences, how they are coped with, and how they interfere with daily life (being aware of their emotional reactivity and behavioral patterns). Identification and naming of the stressors in their parenting role. Distinction between reacting and responding to stress, being aware of their reactivity in those stressful situations and learning to put a stop to it; One-to-one communication dynamic to help the participants to be aware of their own active hearing and their communication patterns, and how a lot of their parenting stressors are related to communication problems. Group sharing of the calendar of positive events and their parenting experience, also connecting their own parenting experiences and the pain and difficulties they have faced with their physical experience. Home practices will be 24 min meditations and a calendar of negative events;
6	Open meditation: Meditation with the five elements: breathing, body, sounds, thoughts as conscious events (differentiating the event from the content), and open presence (receive what there is in the present moment). Group sharing of the calendar of negative events. Incorporation of more silent spaces in the guided meditations. Second communicative dynamic, with a focus on coping strategies focused on the problem and the emotion. The session will finish with an inspirational reading. Home practices will be 24 min meditations, and applying the reactivity versus responding in those situations that are stressors for them. Conscious movements and watching of audiovisual material with a focus on parenting (being aware that parenting involves letting go and giving life for them to be autonomous). Questionnaire to evaluate the feedback of the intervention process;
7	Relating to new experiences: Group dynamic that makes the participants change their sitting place and change their routine, reflecting on how that change influences their thoughts, emotions, and bodily experiences. Transformational coping strategies, attitudes, and behaviors that improve psychological characteristics such as strength in the face of stress or resilience. Group dynamic on relational styles (role-playing with the different relational styles and after group discussion). Emphasis on the internal resources and being able to identify them. Communication dynamic to identify different communication patterns, with participants identifying their own communication styles. Learning of other anchors besides breathing. Questionnaire to evaluate the feedback of the intervention process. Home practices will be 24 min meditations;
8	Strengthening learning (intensive day): Day of silence, application of MBSR-based skills in different life situations. Emphasis on the awareness of the present moment and silent sharing with other parents. Importance of walking meditation. Emphasis on the fact that this day does not necessarily involve a nice experience, and acceptance of this uncomfortable experience;
9	Listen to yourself: Meditation practices in silence without guidance. Discovering inner voices through guided meditations and encouraging personal reflection. Reflection on what the participants want to remember, what they have learned about themselves, and things that they have been emotional about. Writing of a letter with three short-term challenges and long-term challenges, and the possible barriers. Introduction of the positive parenting sessions;
10	Specific positive parenting session: Emphasis on the complexity of the parenting role and reflection on how each person’s own history shapes how they are as parents, working on the awareness of their established patterns as a parent. Attachment and communication along with the promotion of autonomy in their children. Communication dynamics based on listening with full attention, non-judgmental acceptance, and compassion. Additionally, assertive communication strategies, with an emphasis on being aware of the child’s thoughts and feelings and not judging them while talking to them;
11	Specific positive parenting session: Parenting practices related to establishing limits without being authoritarian, adapting those practices to the developmental period of their children and the importance of negotiation, particularly during adolescence. Focus on self-regulation and expectations as key elements to avoid the escalation of possible conflictive situations. Role-playing of emerging situations related to their parenting roles. For example, a common emergent situation would be a child arriving home after curfew. Parents represent those situations and work on how to avoid reacting rather than responding, how to handle their emotions, and how to be aware of their own and their child’s responses. Post-test effectiveness and satisfaction instruments battery and discussion group.

## Data Availability

Data sharing not available due to ethical issues. Consent forms signed by participants did not include data sharing.

## Appendix A

### Appendix A.1. Sociodemographic Profile Questionnaire

**Table A1:** Questions from the sociodemographic profile questionnaire.

**A. THINK ABOUT YOURSELF**	
1. Age	
2. Sex	0. Man 1. Woman
3. Educational level	1. Without studies 2. Primary studies 3. Secondary studies 4. University studies
4. Employment status	1. Housewife or not looking for work 2. Unemployed but looking for work 3. Employed: (a) No qualification required (b) Requires medium qualification (c) Requires high qualification
5. Job stability	0. No 1. Yes
6. Do you have previous experience with meditation or mindfulness?	0. No 1. Yes. (a) Could you describe it? (b) How long?
7. Do you do some kind of activity in your free time that you consider relaxing? (“stress reliever”, for example, sports, painting, cooking dances...)	0. No 1. Yes (a) Which one? (b) How often?
**B. THINK ABOUT YOUR FAMILY**	
1. Type of family	1. Single parent 2. Bi-parental
2. Number of people in the family	
3. Number of children under 18 years old	
4. Number of children under 14 years old	
5. Family stability	0. No 1. Yes
6. Does the family own a car, van or truck?	0. No 1. Yes, one 2. Yes, two or more
7. Does each child have a room?	0. No 1. Yes
8. During the last 12 months, how many times have you traveled on family vacations (at least two nights away from home?	0. None 1. Once 2. Twice 3. More than twice
9. How many computers are there at home? (laptops and tablets are also considered computers)	0. None 1. One 2. Two 3. More than two
**C. THINK ABOUT YOUR CHILD**	
1. Age	
2. Sex	0. Boy 1. Girl
3. Diagnostic category	1. Externalizing disorders 2. Internalizing disorders 3. Others

### Appendix A.2. Feedback Questionnaire of the Intervention Process

**Table A2:** Questions from the feedback questionnaire of the intervention process.

1. What is your assessment of the training so far?
2. Is the training meeting your expectations?
3. Are you learning something useful?
4. How are you feeling in the group?
5. Do you think the level of explanations is appropriate for you?
6. Do you notice changes in your body/mind as a result of these practices? Body scan Mindful yoga movement Meditation
7. What are you doing to solve the difficulties you encounter?
8. Honestly, do you think you spend enough time on home practices?
9. To finish, I invite you to contribute with any suggestion or comment

### Appendix A.3. Field Diary

**Table A3:** Questions from the field diaries.

1. Place where the session takes place	
2. Professional in charge of the group	
3. Which group is it?	
4. Session number	
5. Date	
6. Approximate length of the session	
7. Program activities developed during the session (indicate time)	
8. How was the atmosphere during the development of the session? Indicate whether there has been harmony between the participants during the development of the activities	1. Not adequate 2. Somehow adequate 3. Quite adequate 4. Very adequate
9. What is your general assessment of the sessions?	1-2-3-4-5-6-7-8-9-10
10. Any noticeable issues in the session? Strengths of the program, aspects of the program that have not worked well, or other observations about the course of the session, if there has been any modification in the planning…	
PARTICIPANTS (This information will be provided about every participant after each session)	
1. Participant identifier	Attendance (Yes/No) Level of participation 1. Low 2. Medium 3. High

### Appendix A.4. Follow-Up Questionnaire

**Table A4:** Questions from follow-up questionnaire.

1. What is your assessment of the training after 6 months?
2. What changes/benefits have you noticed in your life? At an individual level In parenting (parent-child relationship and marital relationship, if applicable) In the family environment
3. What mindfulness practices have you incorporated in your daily life?
4. Have you been doing the home practices continuously after the end of the program? 0. No 1. Yes, few times a month 2. Yes, often, weekly 3. Yes, daily
5. What has helped you to incorporate the different practices in your daily life?
6. What barriers have you encountered to incorporate the different practices in your daily life?
7. To finish, I invite you to contribute with any suggestion or comment
